# An Alternative Approach Against Resistent Infections: Long-Term Bacteriophage Therapy in Cystic Fibrosis

**DOI:** 10.3390/v18101050

**Published:** 2026-09-22

**Authors:** Lia Nadareishvili, Nata Nakaidze, Mariam Dadiani, Dea Nizharadze, Elisabed Zaldastanishvili, Nana Balarjishvili, Lika Leshkasheli, Mzia Kutateladze

**Affiliations:** 1 Eliava Phage Therapy Center, 0160 Tbilisi, Georgia; 2 George Eliava Institute of Bacteriophage, Microbiology and Virology, 0160 Tbilisi, Georgia

**Keywords:** bacteriophage therapy, cystic fibrosis, antibiotic-resistance

## Abstract

Cystic fibrosis (CF) is an autosomal recessive genetic disorder that disrupts ion transport across epithelial surfaces, impairing mucociliary clearance and predisposing patients to chronic, often refractory bacterial infections. At the Eliava Phage Therapy Center (EPTC) in Tbilisi, Georgia, we utilize bacteriophage preparations to treat chronic pulmonary infections caused by various bacterial pathogens, including *Staphylococcus aureus*, *Pseudomonas aeruginosa*, *Achromobacter xylosoxidans*, and *Burkholderia cepacia*. This case report describes the successful treatment of a CF-related infection in a male patient with a ΔF508 mutation using Pyophage and Intestiphage preparations, as well as customized phages developed against specific *P. aeruginosa* isolates. The efficacy of phage therapy was assessed by evaluating clinical improvement, pulmonary function, inflammatory markers, and bacterial burden. We observed improvement across all assessed parameters, including a complete eradication of *P. aeruginosa* in sputum culture tests. This case highlights the potential of phage therapy as a primary antimicrobial approach for the long-term management of pulmonary infections in patients with CF.

## 1. Introduction

Cystic Fibrosis (CF) is an autosomal recessive, life-shortening disorder that primarily affects the respiratory, gastrointestinal, and reproductive systems [1]. A mutation in the CFTR gene leads to decreased CFTR protein function and defective mucociliary clearance, which in turn creates an ideal environment for the growth of pathogenic bacteria [2]. Worldwide, more than 70,000 people are affected [3], with the highest prevalence in Northern Europe. In the 1970s, children with CF typically survived to only 5 years of age [4]; however, advances in CF management have since led to substantial improvements in survival and long-term outcomes. According to the 2025 CF Foundation Registry, for people with CF born between 2021 and 2025, the median predicted survival age is 66 years [5].

The prevalence of specific pathogens that cause secondary infections varies among CF patients. Pediatric cases often involve *Staphylococcus aureus* and *Haemophiles influenzae*, while *Pseudomonas aeruginosa* becomes the most commonly diagnosed pathogen in individuals aged 20–30 years [6]. Importantly, *P. aeruginosa* is a major contributor of a decline in lung function and is a leading cause of mortality in this age group [7]. Another significant pathogen in CF patients is *Burkholderia cepacia*, which is prevalent across all age groups and is characterized by rapid lung function decline, increased inflammation, and significant mortality [8]. Associated pulmonary infections are characteristically chronic and refractory, with persistent bacterial colonization and recurrent exacerbations despite prolonged and often intensive antimicrobial therapy.

In patients with CF, pancreatic insufficiency resulting from CFTR dysfunction and obstruction of the pancreatic ducts can impair digestion and nutrient absorption [9]. Consequently, many individuals require pancreatic enzyme replacement therapy, while those who develop CF-related diabetes may also require insulin therapy. Prolonged and repeated antibiotic exposure for chronic pulmonary infections may further disrupt the intestinal microbiota, increasing gastrointestinal disturbances and affecting microbial homeostasis.

Common medications for CF patients include mucolytics, bronchodilators, antimicrobials, anti-inflammatory medications, and CFTR modulator therapies. Despite advances in CF diagnosis and treatment, the spectrum of antimicrobial options remains limited. For patients who have been undergoing long-term antibiotic therapy, often starting during childhood and sometimes involving daily low-dose regimens, the development of resistance is often expected. Antibiotic resistance against *P. aeruginosa* among CF patients is widely observed across multiple antibiotic classes, complicating effective treatment strategies [10].

In response to the development of resistance to antimicrobials, bacteriophage therapy (BT) has reemerged as a focal point, capturing increasing recognition as an alternative treatment for CF associated secondary infections [11,12,13]. Bacteriophages, or phages, are naturally occurring viruses that co-evolve with bacteria. Phages have been studied, developed, and used in clinical practice by the George Eliava Institute of Bacteriophages, Microbiology and Virology, located in Tbilisi, Georgia for more than 100 years. Phage-based preparations developed at the Institute have been successfully used for the treatment of various bacterial complications, including secondary infections in CF patients [14].

BT has shown promising results in animal models. Preclinical studies, such as the one by Morello et al., have verified the efficacy of phage application in the mouse lung infection model with multidrug-resistant *P. aeruginosa* [15].

Several publications show efficacy of phage application in CF patients. Kutateladze and Adamia reported an effective use of a commercially produced bacteriophage cocktail, Pyophage, in the treatment of a 5-year-old patient with *S. aureus* and *P. aeruginosa* co-infections [14]. Hoyle et al. described favorable outcomes in a 17-year-old patient, with an *Achromobacter xylosoxidans* infection awaiting lung transplantation, facilitating a successful surgery [16]. BT can be considered a potential adjunct to antibiotic therapy in pneumonia caused by multidrug-resistant *P. aeruginosa* [17]. Most recently, Hahn et al. used bacteriophages to treat pan-drug-resistant *P. aeruginosa* infections in both pediatric and adult CF patients, reporting symptomatic improvement [18].

The current paper describes a case detailing the successful management of chronic pulmonary infections in a CF patient, who underwent therapy at EPTC from 2017 to 2025. It is a continuation of the case description first provided by Zaldastanishvili et al., in 2021 [19]. Long-term, personalized phage therapy was associated with significant clinical improvement, enhanced quality of life, and complete eradication of the causative agent of secondary infection *P. aeruginosa*, enabling the replacement of conventional antibiotic therapy with phage monotherapy.

## 2. Case Description

A male patient from France, was diagnosed with CF at the age of three (ICD10- E84.0, homozygous gene type delta F508). Starting in childhood, he experienced multiple pulmonary infections involving various bacterial pathogens, including *P. aeruginosa*, for which he received different classes of antibiotics (penicillins, cephalosporins, and macrolides) both orally and intravenously. By the age of 43, he was undergoing maintenance therapy with IV antibiotics twice a year, yet sputum culture tests performed in France indicated the persistent resistance of isolated *P. aeruginosa* to antibiotics.

In pursuit of a more permanent solution, the patient sought assistance from EPTC, exploring the potential benefits of bacteriophage treatment (BT). Since 2017, he has been receiving phage treatment, both locally at our clinic and remotely, in France. From the history of present illness, it is notable that the patient has both exocrine and endocrine pancreatic dysfunction. He has been insulin-dependent for more than 20 years and has received appropriate vitamin supplementation. His diet is well controlled, and his current BMI remains within the normal range. His immunizations are up to date, including three doses of the anti-SARS-CoV-2 vaccine and annual influenza vaccination.

In order to isolate the causative bacterial pathogen, as well as to monitor the improvement of the patient health status, sputum culture tests were performed 13 times at the Eliava Diagnostic Center between 2017 and 2025. The following bacteria were isolated during these tests: *P. aeruginosa*, *Staphylococci* (*aureus*, *chromogenes*, *epidermidis*), *Streptococci* (*oralis*, *mitis*, *salivarius*), *Enterococcus faecalis*, *Enterobacter cloacae* and *Citrobacter koseri.* Among these pathogens, *P. aeruginosa* and *S. aureus* were consistently identified as the predominant clinically relevant pathogens and were associated with the patient’s clinical symptoms. Most samples demonstrated polymicrobial infection.

Phage susceptibility testing was routinely performed for all clinical isolates against the commercially available phage preparations Pyophage and Intestiphage. Initially, the pathogens isolated from the patient’s sputum were susceptible to both preparations; however, phage susceptibility changed over the course of treatment (after 2 years of phage use), with isolates obtained on 15 January 2019, 14 September 2021, and 20 October 2022, demonstrating resistance to both preparations. Customized phage preparations were subsequently developed to target the resistant *P. aeruginosa* strains. The dates of sampling, bacterial loads, and phage susceptibility profiles are presented in Table 1.

During phage therapy, the *P. aeruginosa* bacterial burden progressively decreased from 5 × 10^6^ CFU/mL in 2017 to 1 × 10^5^ CFU/mL in 2021. Subsequent cultures performed in 2022, 2023, and 2024 detected only *S. aureus*, with no recurrence of *P. aeruginosa*. Two independent bacteriological assessments of the sputum samples performed in December 2025—one at the Eliava Diagnostic Center during the patient’s visit, and the other in France—showed no detectable *P. aeruginosa* or *S. aureus*.

## 3. Phage Treatment Evaluation: Changes in Bacterial Burden

The selection of the treatment regimen was guided by the patient’s phagogram (Table 1) and tailored according to the specific clinical complaints. Our strategy involved using Pyophage and Intestiphage—commercially available preparations with predefined composition (initially developed at the Eliava Institute and now manufactured by the Eliava Biopreparations). Following the development of resistance towards these phages, we opted to switch to customized phages, which were selected and developed by the George Eliava Institute of Bacteriophages, Microbiology and Virology. The treatment timeline is presented on Figure 1.

2017: The initial 20-day course consisted of daily oral administration of Pyophage and Intestiphage (8 mL each; phage components with concentration 1 × 10^5^–1 × 10^6^ PFU/mL) and inhalation via a jet nebulizer (2 mL each). After completing the 20-day course, the patient was discharged on 27 January 2017. The treatment was subsequently continued with daily inhalations of Pyophage and Intestiphage throughout the year, supplemented by a 20-day course of oral administration of the same phage preparations every three months.

2018: The same treatment plan was repeated as in 2017.

2019: The patient started therapy with a custom bacteriophage targeting the specific *P. aeruginosa* strain (with concentration 8 × 10^7^ PFU/mL). Subsequent sputum cultures demonstrated early resistance to this preparation, prompting development of a second custom phage. Treatment with the second formulation began in October 2019 and continued into 2020. Phages were administered daily via jet nebulizer, comprising two 20-day courses separated by a two-week interval. Notably, by the end of 2019—following four 20-day courses separated by two-week intervals—the patient’s *P. aeruginosa* isolate again demonstrated susceptibility to Pyophage and Intestiphage.

2020: Pyophage administered by inhalation at a dose of 2 mL twice daily for 3 weeks, followed by a 3-week break—served as the standard protocol. The same dosing schedule, frequency, and cycle duration was applied in all subsequent years, with only the specific phage preparations changing:

2021: New *P. aeruginosa* Custom Phage (concentration of final 1 × 10^8^ PFU/mL).

2022–2023: Pyophage.

2024: Phago Pyo and Phago Staph. (Note: the analogs of Pyophage and Staphylococcal phage with new trade name).

2025: Phago Pyo and Phago Staph.

## 4. Clinical Data Analyses

Prior to bacteriophage therapy, the patient’s chief complaints included frequent productive cough, significant sputum production in the morning, and fatigue upon exertion. Notably, within the initial two weeks of starting the treatment, the patient reported a significant improvement in overall well-being. A substantial reduction in both the frequency of coughing episodes and the volume of sputum production has been observed over the years. Furthermore, there has been a marked increase in energy levels, enabling the patient to engage in regular exercise.

In 2017, during the patient’s initial visit to the EPTC clinic, the recorded vital signs showed a pulse rate (P) of 71′–76′ beats per minute, blood pressure (T/A) of 120/80 mm Hg, and oxygen saturation (%SpO_2_) ranging between 95% and 97%; the respiratory rate (RR) was 26–28 breaths per minute. Pulmonary auscultation revealed the presence of multiple dry and wet crackles, along with bilateral wheezing. There was evidence of hyperemia in the pharyngeal mucosa. Post discharge, %SpO_2_ was 97–98%. By 2025, the patient’s oxygen saturation has shown improvement reaching levels between 98% and 99%; Auscultatory findings indicate positive improvement, characterized by a reduction in crackles and diminished wheezing. There are no signs of oral mucosal inflammation. Spirometry results further support a positive trend, with a notable increase in forced vital capacity (FVC) from 68% in 2017 (pre-treatment) to 76% post-discharge, which was maintained in 2023 and increased further to 79% in 2025.

Furthermore, the levels of C-reactive protein (CRP), a marker of inflammation, have exhibited a marked reduction throughout the course of therapy. In 2017, the patient’s CRP was 13.8 mg/L (pre-treatment), exceeding the typical upper limit of 3.0 mg/L for a healthy adult. Over the course of the therapeutic intervention, a steady decline in CRP was observed: post-discharge CRP was 2.5; by 2023, the level had decreased to 2.32 mg/L, and by 2025 it dropped to 0.75 mg/L.

In October 2023, during the patient’s visit to EPTC, a phage presence (shedding) was evaluated in the patient’s sputum. The testing was performed in a one-month phage-free period, when the patient was not taking phages, as well as after a week-long regimen of combined Intestiphage and Pyophage treatment. None of the samples contained any phage particles.

## 5. Evaluation of Phage-Neutralizing Antibodies

In order to evaluate the level of phage-neutralizing antibodies during the phage treatment, we examined the patient’s serum on four occasions: September 2021, October 2022, September 2023 and December 2025 (Table 2). For the neutralization tests, the phage components of Pyophage targeting *S. aureus*, *P. aeruginosa*, *Proteus* spp., *E. coli* and *Streptococccus* spp. were utilized.

The objective of the study was to determine whether long-term phage therapy induced the production of serum antibodies capable of neutralizing bacteriophages. Four serum samples were diluted 1:50 in bacterial growth medium and mixed with phage suspensions at a serum-to-phage ratio of 9:1, resulting in a final phage concentration of 1 × 10^6^ PFU/mL. After incubation at 37 °C, aliquots were collected at different time points, diluted 100-fold in growth medium, and analyzed using the double-layer agar plaque assay with the corresponding host bacterial strain [21]. Plaque counts were determined for serum-containing and control phage samples, and the percentage of phage neutralization was calculated. All experiments were performed in three biological replicates. The results of the study are summarized in Table 2. The Staphylococcal phage component exhibited consistently high susceptibility to serum-mediated neutralization, reaching nearly complete inhibition (≈100%) after 30 min in all four serum samples, indicating a strong immune response against the Staphylococcal phage component of Pyophage. This likely reflects prior exposure to staphylococcal antigens or strong immunogenic properties to the phage itself.

In contrast, neutralization of the *P. aeruginosa* phage component showed a declining trend over time. While the serum sample from 2021 neutralized 86% of phage particles after 30 min of exposure, this number decreased to 54.0% and 34.6% in the serum samples from 2022 and 2023, respectively. Neutralization was not observed when the serum sample from 2025 was examined. This reduction suggests that the anti-phage humoral response directed against the *P. aeruginosa* phage component diminished during long-term follow-up. The absence of detectable neutralizing activity in 2025 may reflect a progressive decline in circulating neutralizing antibodies due to the lack of continuous antigenic stimulation after successful eradication of *P. aeruginosa*. Such a decline is consistent with the natural contraction of antibody responses following clearance of the target antigen, particularly when repeated exposure is absent.

No neutralization was observed for phages against *Proteus*, *E. coli* and *Streptococci*.

## 6. Discussion

It should be recognized that a significant portion of the phage treatment of the patient was carried out in France. In general, remote treatment creates limitations in setting a rigid treatment timeline and assessing everyday progress of the patient. We relied on the patient report of whether he had received phages according to the instructions given by the EPTC doctors. Despite these limitations, we had the opportunity to observe a gradual improvement of this patient since 2017, by assessing his health status annually, providing a solid foundation to claim the efficacy of BT.

In the context of CF, *P. aeruginosa* exhibits marked resistance to a broad spectrum of antibiotics, attributable to both intrinsic as well as acquired mechanisms of resistance [22]. Biofilms formation protects *P. aeruginosa* from the host’s immune system as well as makes it extremely resilient to antimicrobial treatment [23]. In case of our patient, we have reached complete eradication of the infection, and empiric antibiotics have been successfully replaced by bacteriophages. The potential of personalized bacteriophage therapy to overcome one of the greatest therapeutic challenges in CF—the management of chronic, refractory infections—while reducing dependence on long-term antibiotic treatment should be further explored.

Management of CF requires a combination of strategies to control infection, reduce inflammation, and improve pulmonary function. Bacteriophage therapy has emerged as a promising approach to target persistent bacterial pathogens such as *P. aeruginosa*, while CFTR modulators like Kaftrio (Elexacaftor/Tezacaftor/Ivacaftor) address the underlying chloride transport defect, improving mucus clearance and overall respiratory function. Following the successful eradication of *P. aeruginosa*, the patient began Kaftrio therapy in 2022. He is currently taking Trikafta, the same combination therapy marketed under a different name.

It has been hypothesized that Kaftrio use can significantly decrease the frequency of IV antibiotic use in CF patients [24]. The study by Volkova et al. analyzed data from national cystic fibrosis registries in the United States and the United Kingdom to assess the impact of Ivacaftor treatment on disease progression in patients with cystic fibrosis [25]. Importantly, the study observed that the prevalence of *P. aeruginosa* decreased in the group of patients that received CFTR modulator treatment, thus lowering respiratory microbiome burden. Furthermore, CFTR modulators increase the life expectancy of patients. As an example, in Poland where such treatment is not available, the average life expectancy remains to be 24.5 years, compared to 46 years in the United States [26]. In further studies, it will be interesting to see the role of phage preparations in combination with CFTR modulators.

Torres-Barceló and Hochberg argue that combining bacteriophage therapy with antibiotics can improve treatment outcomes and limit antibiotic resistance [27]. They suggest that phages, which specifically target bacterial cells, may complement traditional antibiotics in managing chronic infections. However, the authors emphasize the need to understand the interactions between phages, bacteria, and the host, and to select therapeutic agents carefully to maximize efficacy and minimize resistance. In our practice, whether phages are used alone or in combination with antibiotics depends on the severity of the patient’s symptoms. It is also worth mentioning that phages have the potential to “modify” antibiotic resistance, as well as to reduce the minimal inhibitory concentration of used antibiotics, thus enhancing antibiotic activity [28,29,30,31], which is another advantage of a combined use.

Bacterial resistance to bacteriophages is a concern in the field of BT [32,33,34,35]. Over time, bacteria can evolve mechanisms to resist phage infection (mainly, in case of mono-phage use), which will reduce the effectiveness of the therapy. Some of the mechanisms of resistance include receptor adaptations, bacterial host defense systems, nucleic acid interference, abortive infection, and chemical defense [36]. Regarding the clinical case described above, we have observed the emergence of resistance to Pyophage and Intestiphage (mixtures of several different phage components) after prolonged (two years) regular use. Since the problem of resistance could impede the treatment process with bacteriophages, it is essential to minimize possibilities for development of phage-resistance—instead of mono-phage, use of a mixture of different phages that target the same bacteria.

The formation of phage-neutralizing antibodies may negatively impact the outcome of phage therapy, especially during long-term use. The development of antibodies against certain bacteriophages indicates that this process is phage-specific and temporally dynamic, with strong, stable responses against certain phages and declining or minimal responses against others. The absence of detectable neutralizing antibodies against the *Pseudomonas* phage despite long-term bacterial infection likely reflects phage-specific immune recognition. This may be explained by the low immunogenicity of the phage. As for the high index of neutralization against Staphylococcal phages, our observation is consistent with the results described by several medical doctors [37]. Naturally occurring phage-neutralizing antibodies against Staphylococcal phages have been detected in healthy individuals without prior exposure to phage therapy. However, in patients treated with phages, the clinical outcome and efficacy of phage therapy are not affected by the presence of high concentrations of phage-specific antibodies.

The microbiome in cystic fibrosis patients is increasingly recognized as a key factor in disease progression [38]. While traditional CF microbiology focused on a limited set of opportunistic bacterial pathogens, recent studies reveal a complex and diverse microbial community in the airways and gastrointestinal tract. Early and repeated antibiotic use can alter normal microbiome, influencing overall patient health. Bacteriophage therapy offers a major advantage by specifically targeting pathogenic bacteria without disrupting normal microflora, helping to maintain microbial balance and gastrointestinal homeostasis. In our clinical practice, we administer phage therapy to patients with CF both orally and via nebulization. This combined approach has shown benefits in the treatment of pulmonary infections and has also contributed to improvements in gastrointestinal symptoms associated with CF.

The biggest challenge facing phage therapy is conducting successful, well-controlled large-scale prospective clinical trials. Such trials are the cornerstone of evidence-based medicine and are essential for determining the safety and efficacy of any medical intervention; also, trials are key elements for wider, global legal approval of phage-based preparations as effective antibacterial medication. Since CF is a genetic disease that starts progressing at a pediatric age, the validity of the results should be tested in pediatric patients and adult patients separately. Further clinical trials should address questions regarding the most effective route of administration, standardization of delivery methods, combined use of antibiotics, and other related factors [39]. It is also important to evaluate the comparative efficacy of monophage therapy versus phage cocktail treatments [40].

Armata Pharmaceuticals reported positive results for an inhaled bacteriophage therapy targeting *P. aeruginosa* in cystic fibrosis patients, from Phase 1b/2a study. The therapy was well tolerated with a safety profile comparable to placebo. Pharmacokinetic analysis confirmed effective lung delivery and minimal systemic exposure. Notably, patients with higher phage exposure showed a sustained reduction in bacterial load, supporting further clinical development [41]. Scientific society and medical communities as well as patients are looking forward to reviewing the results of several clinical trials performed in different countries on phage therapy for the treatment of cystic fibrosis. Reliable results would help the global understanding and acceptance of bacteriophage treatment of different infectious diseases, including secondary infections associated with various serious medical complications, such as cystic fibrosis.

## 7. Conclusions

Our clinical experience in treating CF patients, including a detailed analysis of this case, leads us to believe that bacteriophages would be a valuable addition to the antimicrobial options for chronic pulmonary infections, offering a potential alternative or adjuvant to antibiotics. In this patient, we achieved the following treatment goals: improvement of clinical symptoms, enhanced spirometry results, and a decrease in inflammatory markers. Most importantly, we achieved complete eradication of the *P. aeruginosa* infection, which persisted up until the time of writing of this case report.

## Figures and Tables

**Figure 1:**
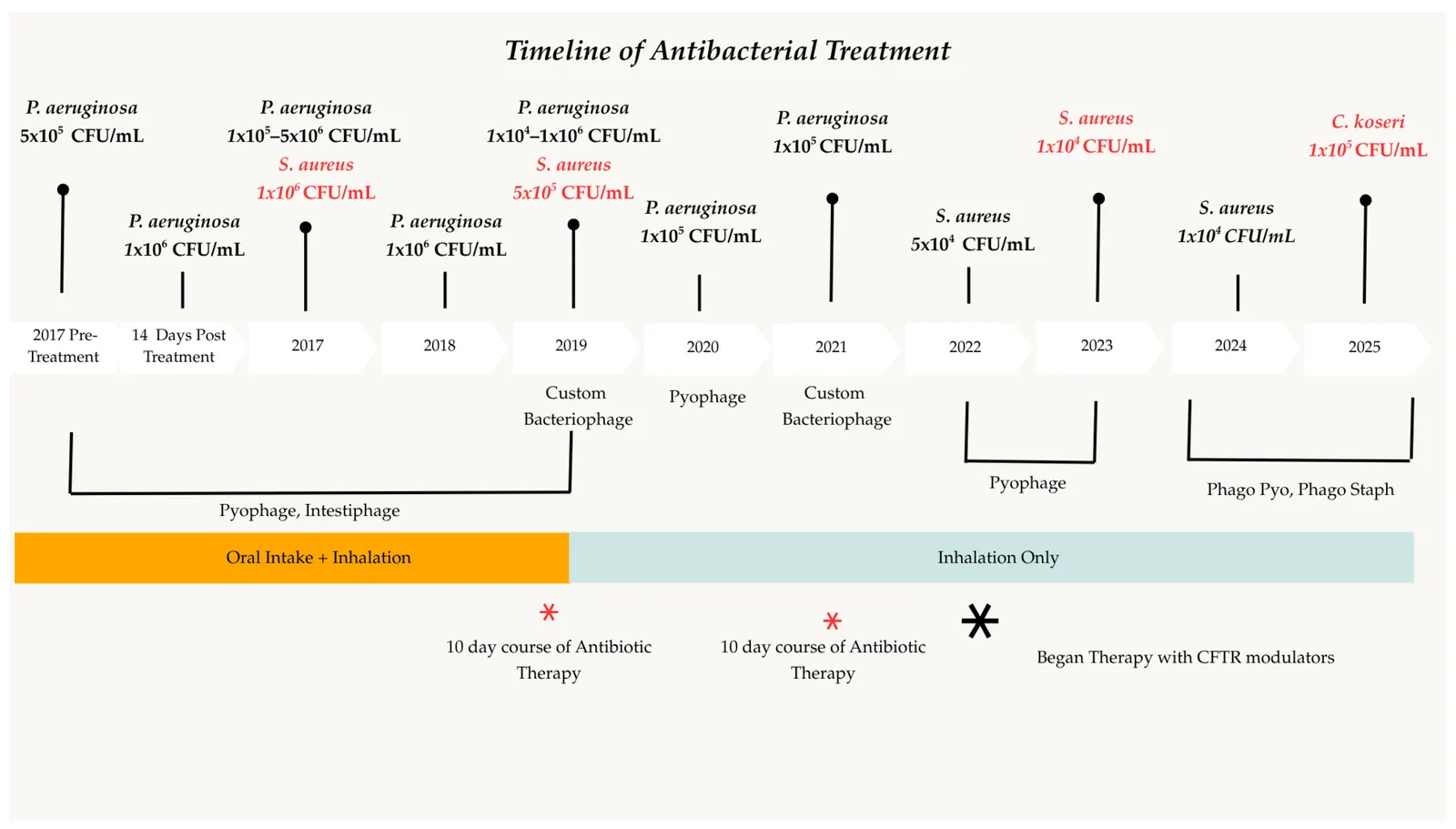
The figure demonstrates bacterial burden in clinical samples and phage therapy timeline.

**Table 1:** Sputum culture tests performed at the Eliava Diagnostic Center from 2017 to 2025.

Sputum Culture Collection Date	Isolated Pathogens	Susceptibility to Pyophage	Susceptibility to Intestiphage
9 January 2017	*P. aeruginosa* 5 × 10^5^ CFU/mL	S	S
24 January 2017	*P. aeruginosa* 1 × 10^6^ CFU/mL	S	S
3 April 2017	*P. aeruginosa* 1 × 10^5^ CFU/mL *S. aureus* 1 × 10^6^ CFU/mL	I	I
23 September 2017	*P. aeruginosa* 5 × 10^6^ CFU/mL	I	I
17 September 2018	*P. aeruginosa* 1 × 10^6^ CFU/mL	I	I
15 January 2019	*P. aeruginosa* 1 × 10^6^ CFU/mL	R	R
1 October 2019	*P. aeruginosa* 1 × 10^4^ CFU/mL	I	I
	*S. aureus* 5 × 10^5^ CFU/mL	S	S
23 September 2020	*P. aeruginosa* 1 × 10^5^ CFU/mL	I	I
14 September 2021	*P. aeruginosa* 1 × 10^5^ CFU/mL	R	R
20 October, 2022	*S. aureus* 5 × 10^4^ CFU/mL	R	R
8 September 2023	*S. aureus* 1 × 10^4^ CFU/mL	S	S
2 August 2024	*S. aureus* 1 × 10^4^ CFU/mL	I	I
9 December, 2025	*C. koseri* 1 × 10^5^ CFU/mL	N/A	N/A

Note: S—susceptible, I—intermediate reaction, R—resistant, and N/A—not-applicable [20].

**Table 2:** Phage neutralization results (in %) in patient’s serum samples.

Components of Pyo Phage	After 10 min				After 30 min			
	Serum 1, 2021 September	Serum 2, 2022 October	Serum 3, 2023 September	Serum4, 2025 December	Serum 1, 2021 September	Serum 2, 2022 October	Serum 3, 2023 September	Serum 4, 2025 December
*S. aureus*	99.9	93.2	99.7	100	100	99.8	100	100
*P. aeruginosa*	53.9	27.1	11.3	0	86.0	54.0	34.6	0
*Proteus* spp.	0	0	2.4	0	0	0	0	0
*E. coli*	0	0	0	0	0	0	0	0
*Streptococcus* spp.	0	0	-	-	0	0	-	-

## Data Availability

Data is unavailable due to privacy restrictions.

## References

[1] Chen Q., Shen Y., Zheng J. (2021). A review of cystic fibrosis: Basic and clinical aspects. Anim. Models Exp. Med..

[2] Françoise A., Héry-Arnaud G. (2020). The microbiome in cystic fibrosis pulmonary disease. Genes.

[3] Sepahzad A., Morris-Rosendahl D.J., Davies J.C. (2021). Cystic fibrosis lung disease modifiers and their relevance in the new era of precision medicine. Genes.

[4] Fanen P., Wohlhuter-Haddad A., Hinzpeter A. (2014). Genetics of cystic fibrosis: CFTR mutation classifications toward genotype-based CF therapies. Int. J. Biochem. Cell Biol..

[5] Cystic Fibrosis Foundation (2026). 2025 Cystic Fibrosis Foundation Patient Registry Highlights. https://www.cff.org/medical-professionals/2025-patient-registry-highlights.

[6] Coutinho H.D., Falcão-Silva V.S., Gonçalves G.F. (2008). Pulmonary bacterial pathogens in cystic fibrosis patients and antibiotic therapy: A tool for the health workers. Int. Arch. Med..

[7] Lyczak J.B., Cannon C.L., Pier G.B. (2002). Lung infections associated with cystic fibrosis. Clin. Microbiol. Rev..

[8] Canning J.S., Laucirica D.R., Ling K.-M., Nicol M.P., Stick S.M., Kicic A. (2024). Phage therapy to treat cystic fibrosis *Burkholderia cepacia* complex lung infections: Perspectives and challenges. Front. Microbiol..

[9] Gibson-Corley K.N., Meyerholz D.K., Engelhardt J.F. (2016). Pancreatic pathophysiology in cystic fibrosis. J. Pathol..

[10] Bonyadi P., Saleh N.T., Dehghani M., Yamini M., Amini K. (2022). Prevalence of antibiotic resistance of *Pseudomonas aeruginosa* in cystic fibrosis infection: A systematic review and meta-analysis. Microb. Pathog..

[11] Cocorullo M., Stelitano G., Chiarelli L.R. (2024). Phage therapy: An alternative approach to combating multidrug-resistant bacterial infections in cystic fibrosis. Int. J. Mol. Sci..

[12] Ling K.-M., Stick S.M., Kicic A. (2023). Pulmonary bacteriophage and cystic fibrosis airway mucus: Friends or foes?. Front. Med..

[13] Hitchcock N.M., Devequi Gomes Nunes D., Shiach J., Valeria Saraiva Hodel K., Dantas Viana Barbosa J., Alencar Pereira Rodrigues L., Coler B.S., Botelho Pereira Soares M., Badaró R. (2023). Current clinical landscape and global potential of bacteriophage therapy. Viruses.

[14] Kutateladze M., Adamia R. (2008). Phage therapy experience at the Eliava Institute. Med. Mal. Infect..

[15] Morello E., Saussereau E., Maura D., Huerre M., Touqui L., Debarbieux L. (2011). Pulmonary bacteriophage therapy on *Pseudomonas aeruginosa* cystic fibrosis strains: First steps towards treatment and prevention. PLoS ONE.

[16] Hoyle N., Zhvaniya P., Balarjishvili N., Bolkvadze D., Nadareishvili L., Nizharadze D., Wittmann J., Rohde C., Kutateladze M. (2018). Phage therapy against *Achromobacter xylosoxidans* lung infection in a patient with cystic fibrosis: A case report. Res. Microbiol..

[17] Law N., Logan C., Yung G., Furr C.L., Lehman S.M., Morales S., Rosas F., Gaidamaka A., Bilinsky I., Grint P. (2019). Successful adjunctive use of bacteriophage therapy for treatment of multidrug-resistant *Pseudomonas aeruginosa* infection in a cystic fibrosis patient. Infection.

[18] Hahn A., Sami I., Chaney H., Koumbourlis A.C., Del Valle Mojica C., Cochrane C., Chan B.K., Koff J.L. (2023). Bacteriophage therapy for pan-drug-resistant *Pseudomonas aeruginosa* in two persons with cystic fibrosis. J. Investig. Med. High Impact Case Rep..

[19] Zaldastanishvili E., Leshkasheli L., Dadiani M., Nadareishvili L., Askilashvili L., Kvatadze N., Goderdzishvili M., Kutateladze M., Balarjishvili N. (2021). Phage therapy experience at the Eliava Phage Therapy Center: Three cases of bacterial persistence. Viruses.

[20] Clokie M.R.J., Kropinski A.M. (2009). Bacteriophages: Methods and Protocols, Volume 1: Isolation, Characterization, and Interactions.

[21] Adams M.H. (1959). Methods of Study of Bacterial Viruses. Bacteriophages.

[22] Pang Z., Raudonis R., Glick B.R., Lin T.J., Cheng Z. (2019). Antibiotic resistance in *Pseudomonas aeruginosa*: Mechanisms and alternative therapeutic strategies. Biotechnol. Adv..

[23] Maurice N.M., Bedi B., Sadikot R.T. (2018). *Pseudomonas aeruginosa* biofilms: Host response and clinical implications in lung infections. Am. J. Respir. Cell Mol. Biol..

[24] Kafka S. (2023). P20 Does Kaftrio^®^ reduce IV antibiotic burden in cystic fibrosis? A retrospective observational evaluation. Arch. Dis. Child..

[25] Volkova N., Moy K., Evans J., Campbell D., Tian S., Simard C., Higgins M., Konstan M.W., Sawicki G.S., Elbert A. (2020). Disease progression in patients with cystic fibrosis treated with ivacaftor: Data from national US and UK registries. J. Cyst. Fibros..

[26] Guo J., Garratt A., Hill A. (2022). Worldwide rates of diagnosis and effective treatment for cystic fibrosis. J. Cyst. Fibros..

[27] Torres-Barceló C., Hochberg M.E. (2016). Evolutionary rationale for phages as complements of antibiotics. Trends Microbiol..

[28] Leshkasheli L., Kusradze I., Bolkvadze D., Askilashvili L., Chichashvili M., Tsertsvadze G., Zaldastanishvili E. (2025). Klebsiella pneumoniae Phage M198 and Its Therapeutic Potential. Viruses.

[29] Diallo K., Dublanchet A. (2022). Benefits of combined phage-antibiotic therapy for the control of antibiotic-resistant bacteria: A literature review. Antibiotics.

[30] Tagliaferri T.L., Jansen M., Horz H.P. (2019). Fighting pathogenic bacteria on two fronts: Phages and antibiotics as combined strategy. Front. Cell. Infect. Microbiol..

[31] Holger D.J., Lev K.L., Kebriaei R., Morrisette T., Shah R., Alexander J., Lehman S.M., Rybak M.J. (2022). Bacteriophage-antibiotic combination therapy for multidrug-resistant *Pseudomonas aeruginosa*: In vitro synergy testing. J. Appl. Microbiol..

[32] Labrie S.J., Samson J.E., Moineau S. (2010). Bacteriophage resistance mechanisms. Nat. Rev. Microbiol..

[33] Khan A.A., Manzoor K.N., Sultan A., Saeed M., Rafique M., Noushad S., Talib A., Rentschler S., Deigner H.-P. (2021). Pulling the Brakes on Fast and Furious Multiple Drug-Resistant (MDR) Bacteria. Int. J. Mol. Sci..

[34] Mangalea M.R., Duerkop B.A. (2020). Fitness Trade-Offs Resulting from Bacteriophage Resistance Potentiate Synergistic Antibacterial Strategies. Infect. Immun..

[35] Hatfull G.F., Dedrick R.M., Schooley R.T. (2022). Phage Therapy for Antibiotic-Resistant Bacterial Infections. Annu. Rev. Med..

[36] Egido J.E., Costa A.R., Aparicio-Maldonado C., Haas P.J., Brouns S.J.J. (2022). Mechanisms and clinical importance of bacteriophage resistance. FEMS Microbiol. Rev..

[37] Kaźmierczak Z., Majewska J., Miernikiewicz P., Międzybrodzki R., Nowak S., Harhala M., Lecion D., Kęska W., Owczarek B., Ciekot J. (2021). Immune Response to Therapeutic Staphylococcal Bacteriophages in Mammals: Kinetics of Induction, Immunogenic Structural Proteins, Natural and Induced Antibodies. Front. Immunol..

[38] Huang Y.J., LiPuma J.J. (2016). The microbiome in cystic fibrosis. Clin. Chest Med..

[39] Hraiech S., Brégeon F., Rolain J.M. (2015). Bacteriophage-based therapy in cystic fibrosis-associated *Pseudomonas aeruginosa* infections: Rationale and current status. Drug Des. Dev. Ther..

[40] Chan B.K., Stanley G., Modak M., Koff J.L., Turner P.E. (2021). Bacteriophage therapy for infections in CF. Pediatr. Pulmonol..

[41] Armata Pharmaceuticals, Inc. (2023). Armata Pharmaceuticals Announces Positive Topline Data from Phase 1b/2a SWARM-P.a. Clinical Trial of Inhaled AP-PA02 in Patients with Cystic Fibrosis. https://www.prnewswire.com/news-releases/armata-pharmaceuticals-announces-positive-topline-data-from-phase-1b2a-swarm-pa-clinical-trial-of-inhaled-ap-pa02-in-patients-with-cystic-fibrosis-301762961.html.

